# Neutrophil extracellular traps are involved in enhanced contact hypersensitivity response in IL-36 receptor antagonist-deficient mice

**DOI:** 10.1038/s41598-022-16449-z

**Published:** 2022-08-04

**Authors:** Yurie Hasegawa, Yohei Iwata, Hidehiko Fukushima, Yoshihito Tanaka, Soichiro Watanabe, Kenta Saito, Hiroyuki Ito, Mizuki Sugiura, Masashi Akiyama, Kazumitsu Sugiura

**Affiliations:** 1 Department of Dermatology, Fujita Health University School of Medicine, 1-98 Dengakugakubo, Kutsukake-cho, Toyoake, Aichi 470-1192 Japan; 2 Department of Dermatology, Nagoya University Graduate School of Medicine, 65 Tsurumai-cho, Showa-ku, Nagoya, Aichi 466-8550 Japan

**Keywords:** Innate immunity, Immunology, Inflammation

## Abstract

Loss-of-function homozygous or compound heterozygous mutations in *IL36RN*, which encodes interleukin-36 receptor antagonist (IL-36Ra), have been implicated in the pathogenesis of skin disorders. We previously reported that *Il36rn*^*−/−*^ mice exhibit an enhanced contact hypersensitivity (CHS) response through increased neutrophil recruitment. In addition, *Il36rn*^*−/−*^ mice show severe imiquimod-induced psoriatic skin lesions and enhanced neutrophil extracellular trap (NET) formation. We hypothesized that NETs may play an important role in the CHS response. To confirm this, we examined the CHS response and NET formation in *Il36rn*^*−/−*^ mice. *Il36rn*^*−/−*^ mice showed enhanced CHS responses, increased infiltration of inflammatory cells, including neutrophils, CD4^+^ T cells, and CD8^+^ T cells, NET formation, and enhanced mRNA expression of cytokines and chemokines, including IL-1β, C-X-C motif chemokine ligand (CXCL)1, CXCL2, and IL-36γ. Furthermore, NET formation blockade improved the CHS response, which consequently decreased inflammatory cell infiltration and NET formation. Consistently, we observed decreased expression of these cytokines and chemokines. These findings indicate that IL-36Ra deficiency aggravates the CHS response caused by excessive inflammatory cell recruitment, NET formation, and cytokine and chemokine production, and that NET formation blockade alleviates the CHS response. Thus, NET formation may play a prominent role in the CHS response.

## Introduction

*IL36RN* encodes interleukin (IL)-36 receptor antagonist (IL-36Ra), a protein of the IL-1 cytokine family, which closely regulates IL-36 signaling^1,2^. In the IL-36 pathway, IL-36α, β, and γ are utilized as specific receptors^1,2^. The IL-36 pathway is activated when IL-36α, β, and γ bind to their specific receptor, interleukin-1 receptor-related protein 2 (IL-1Rrp2), thereby inducing the recruitment of the co-receptor IL-1 receptor accessory protein (IL-1RacP) and activation of the nuclear factor kappa B (NF-κB) and mitogen-activated protein kinase signaling pathways^1,2^. This stimulates the transcription and secretion of pro-inflammatory cytokines^1,2^ and leads to the recruitment of neutrophils, T cells, and dendritic cells (DCs) to the dermis. IL-36 stimulates the production of chemotactic substances by activating leukocytes and promoting leukocyte infiltration and skin acanthosis^3^.

Homozygous or compound heterozygous mutations in the *IL36RN* gene have been implicated in psoriasis-related pustular eruptions, such as generalized pustular psoriasis, acrodermatitis continua of Hallopeau, acute generalized exanthematous pustular eruptions, and palmoplantar pustular psoriasis^4^. Loss-of-function mutations in *IL36RN* cause “deficiency of interleukin-36 receptor antagonist”, a recessively inherited autoinflammatory keratinization disease^5–12^. According to information from the Human Genetic Variation Database, two *IL36RN* founder mutations (c.28 C > T (p. Arg10X) and c.115 + 6 T > C (p. ArgfsX1)) have been detected in less than 2% of the Japanese population^13^. Consequently, a few Japanese individuals carry *IL36RN* mutations, which may have etiological roles in several diseases, including generalized pustular psoriasis.

We previously generated *Il36rn*^*−/−*^ mice^12^ and reported that contact hypersensitivity (CHS) responses are enhanced owing to the increase in neutrophil mobilization with the increase in the generation of CD4^+^ T and CD8^+^ T cells^14^. Furthermore, we previously found that imiquimod-induced psoriatic skin lesions were more severe and that neutrophil extracellular trap (NET) formation was enhanced in *Il36rn*^*−/−*^ mice^15^. However, the immunological pathophysiology of NETs in CHS remains unclear. We hypothesized that NETs play a prominent role in the CHS response in the presence of a high number of CD4^+^ T cells and CD8^+^ T cells. To confirm this possibility, we evaluated NET formation qualitatively or quantitatively in *Il36rn*^*−/−*^ and wild-type mice under the influence of CD4^+^ and CD8^+^ T cells in the CHS response.

## Results

### Estimation of the thickness of the ear in ***Il36rn***^***−/−***^ and wild-type mice treated with Cl-amidine during the CHS response

**Figure 1:**
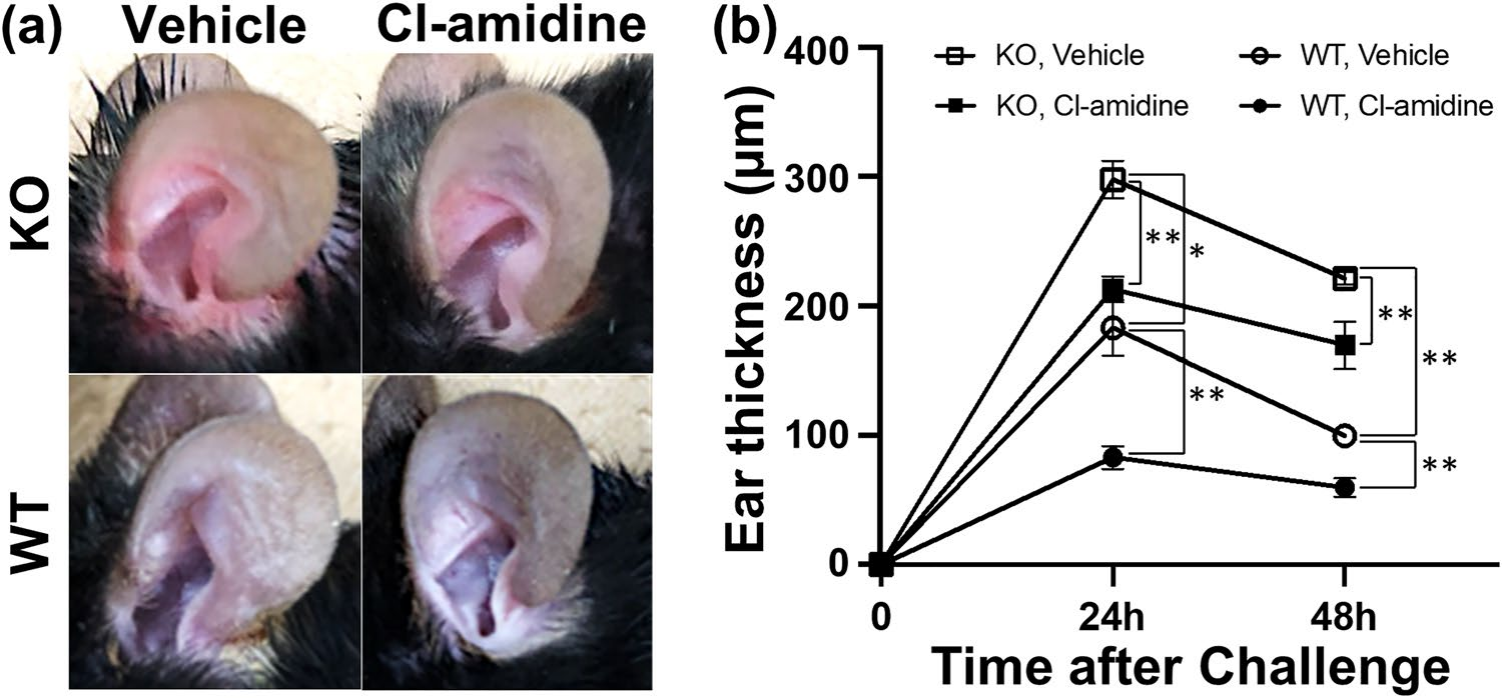
Effect of Cl-amidine on the contact hypersensitivity response in wild-type mice (WT) and *Il36rn*^*−/−*^ mice (KO). (**a**) Representative images of the ear tissues of KO and WT treated with Cl-amidine (10 mg/kg/day) or vehicle 48 h after the DNFB challenge. (**b**) Measurement of ear thickness in KO and WT treated with Cl-amidine (10 mg/kg/day) or vehicle 24 h and 48 h after the DNFB challenge. All values represent the mean ± SEM obtained from six mice per group. (*p < 0.05, ** *p* < 0.01).

First, we evaluated the effect of Cl-amidine on the CHS response in *Il36rn*^*−/−*^ and wild-type mice (Fig. 1). Cl-amidine, a pan-peptidyl arginine deiminase (PAD) inhibitor, subdues the formation of NETs by suppressing PAD4, an enzyme necessary for NET formation^16^. In agreement with findings from previous reports^14^, the CHS response in *Il36rn*^*−/−*^ mice was enhanced in comparison with that in wild-type mice both at 24 h (162.73%; *p < 0.05) and 48 h (221.7%; **p < 0.01) (Fig. 1b). Interestingly, the CHS response was attenuated by Cl-amidine treatment at 24 and 48 h in *Il36rn*^*−/−*^ and wild-type mice in comparison with that in the vehicle control (*Il36rn*^*−/−*^ mice; 28.49% decrease (24 h) **p < 0.01, 23.32% decrease (48 h) **p < 0.01; wild-type mice; 54.56% decrease (24 h) **p < 0.01, 20% decrease (48 h) **p < 0.01) (Fig. 1b). Similar results were obtained in male mice (data not shown). Thus, the CHS response was significantly enhanced by IL-36Ra deficiency and was suppressed in response to Cl-amidine treatment in mice with or without IL-36Ra deficiency.

### Recruitment of inflammatory cell in *Il36rn*^*−/−*^ and wild-type mice during CHS response

**Figure 2:**
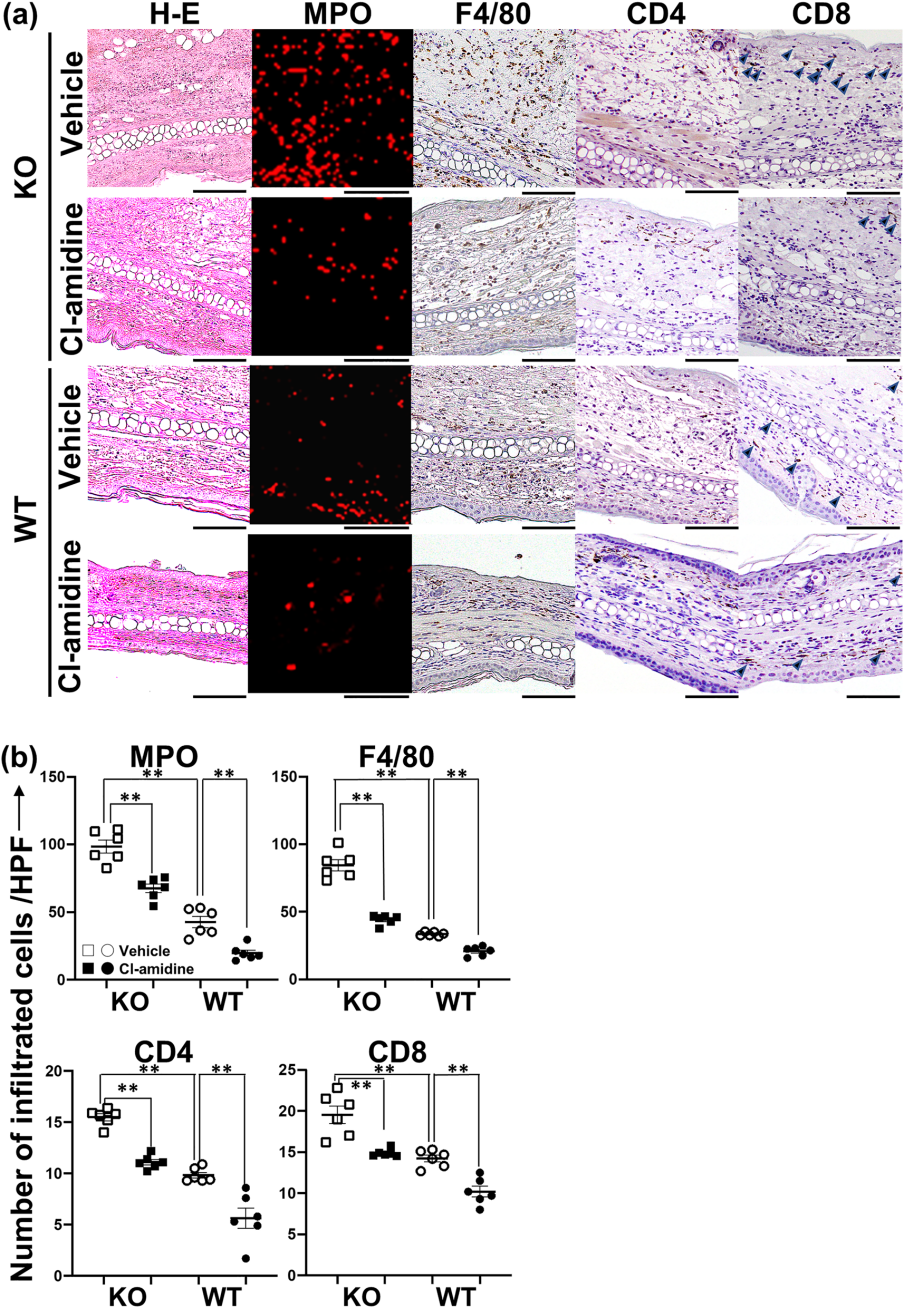
Effect of Cl-amidine on inflammatory cell infiltration in the ear skin during the contact hypersensitivity response in wild-type mice (WT) and *Il36rn*^*−/−*^ mice (KO). (**a**) Representative histological images of ear skin from KO and WT treated with Cl-amidine (10 mg/kg/day) or the same quantity of vehicle 48 h after the DNFB challenge (hematoxylin and eosin (H&E) staining, myeloperoxidase (MPO) staining, F4/80 staining, CD4 staining, and CD8 staining; all scale bars = 100 μm). The infiltrated neutrophils tested positive for MPO (red). The infiltrated macrophages were stained for F4/80. CD8+-positive T cells were indicated by arrow heads. (**b**) The number of MPO^+^ neutrophils (MPO), F4/80^+^ macrophages (F4/80), CD4^+^ T cells (CD4), and CD8^+^ T cells (CD8) per field (0.16 mm^2^) was counted under a microscope. All values represent the mean ± SEM obtained from six mice per group. (**p < 0.01).

Next, we examined inflammatory cell recruitment in the ear tissue of *Il36rn*^*−/−*^ and wild-type mice during the CHS response (Fig. 2). At 48 h after the challenge, we observed a significantly higher number of myeloperoxidase (MPO)-positive neutrophils in *Il36rn*^*−/−*^ mice than in wild-type mice (**p < 0.01). Interestingly, the number of MPO-positive neutrophils significantly decreased upon Cl-amidine treatment in both *Il36rn*^*−/−*^ (**p < 0.01) and wild-type mice (**p < 0.01) (Fig. 2a,b). Similarly, the numbers of F4/80-positive macrophages, CD4^+^ T cells, and CD8^+^ T cells were significantly higher in *Il36rn*^*−/−*^ mice than in wild-type mice (**p < 0.01 for all). The number of F4/80-positive macrophages, CD4^+^ T cells, and CD8^+^ T cells decreased upon Cl-amidine treatment in both *Il36rn*^*−/−*^ mice (**p < 0.01 for all) and wild-type mice (**p < 0.01 for all) (Fig. 2a,b). Thus, Cl-amidine inhibited inflammatory cell recruitment during the CHS response in both *Il36rn*^*−/−*^ and wild-type mice. To investigate whether Cl-amidine has a neutropenic effect, we reacted Cl-amidine with neutrophils in vitro and found that the number of neutrophils did not decrease, indicating that Cl-amidine itself has no neutropenic effect (see Supplementary Fig. S1a,b online).

### NET formation in *Il36rn*^*−/−*^ and wild-type mice during CHS response

**Figure 3:**
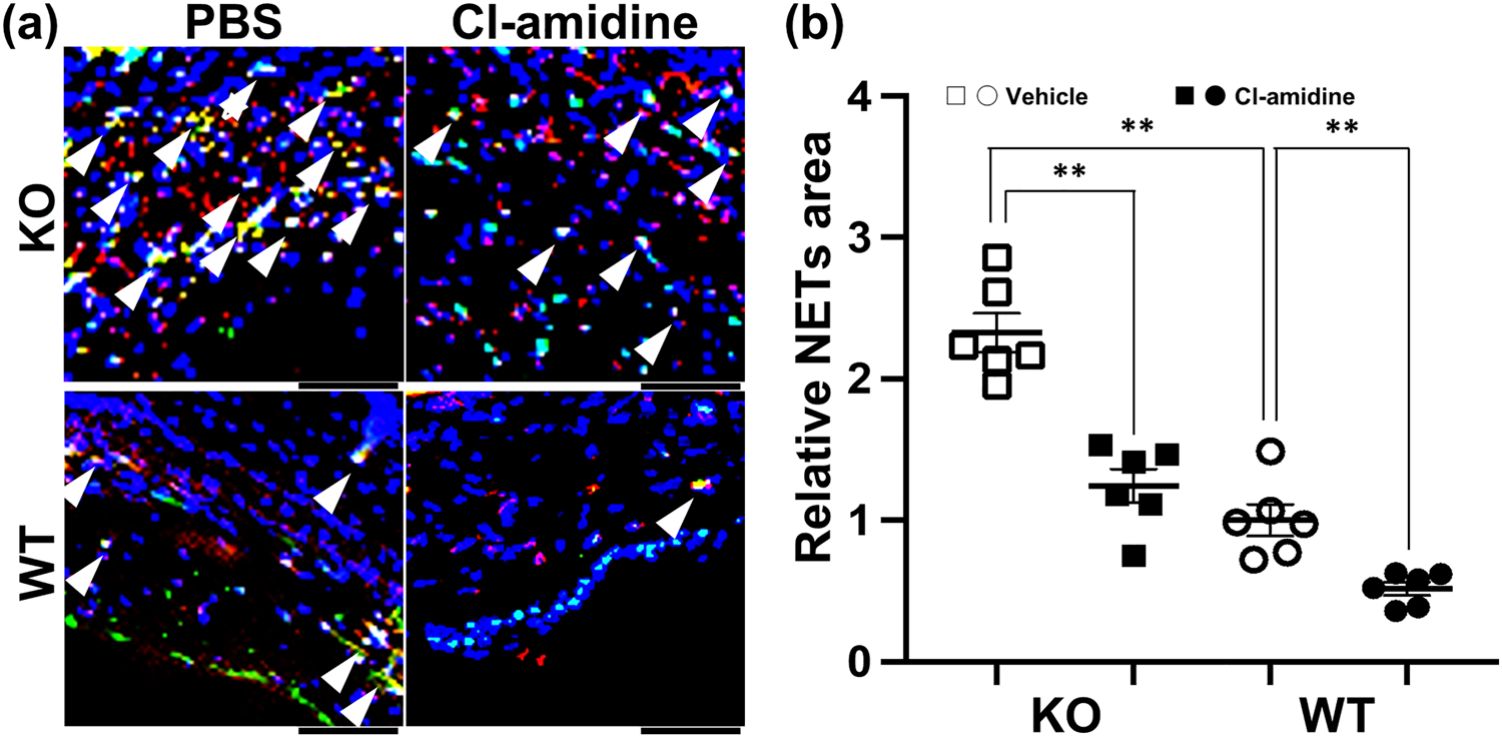
The effect of Cl-amidine on neutrophil extracellular trap (NET) formation during the contact hypersensitivity response in wild-type mice (WT) and *Il36rn*^*−/−*^ mice (KO). (**a**) Representative immunofluorescence image of NET structures. Arrow heads show the formation NETs that were myeloperoxidase-positive (red) and citrullinated histone-positive (green). The nuclei were counterstained with 4’,6-diamidino-2-phenylindole (DAPI; blue). Scale bar = 50 μm. (**b**) The area of NETs was measured using the ImageJ software. Each histogram shows the relative area of NETs at 48 h after the DNFB challenge. All values represent the mean ± SEM obtained from six mice per group (**p < 0.01).

Next, using immunofluorescence staining, we investigated NET formation in the ear tissues during the CHS response (Fig. 3a). The area of NETs in the ear tissues of *Il36rn*^*−/−*^ mice at 48 h after the challenge increased significantly in comparison with that in wild-type mice (324.7 ± 19.32 vs. 139.5 ± 15.55, **p < 0.01) (Fig. 3a,b). Furthermore, NET formation was inhibited in response to Cl-amidine treatment in both *Il36rn*^*−/−*^ mice (324.7 ± 19.32 (vehicle) vs. 173.2 ± 16.63 (Cl-amidine), **p < 0.01) and wild-type mice (139.5 ± 15.55 (vehicle) vs. 71.80 ± 6.675 (Cl-amidine), **p < 0.01) (Fig. 3a,b). Consequently, IL-36Ra deficiency boosted the formation of NETs during the CHS response, whereas Cl-amidine suppressed the formation of NETs in both *Il36rn*^*−/−*^ and wild-type mice. Furthermore, in vitro, Cl-amidine also reduced NET formation, consistent with the in vivo results (see Supplementary Fig. S1c,d online).

### Cytokine and chemokine expression in the ear tissues of *Il36rn*^*−/−*^ mice and wild-type mice treated with Cl-amidine

**Figure 4:**
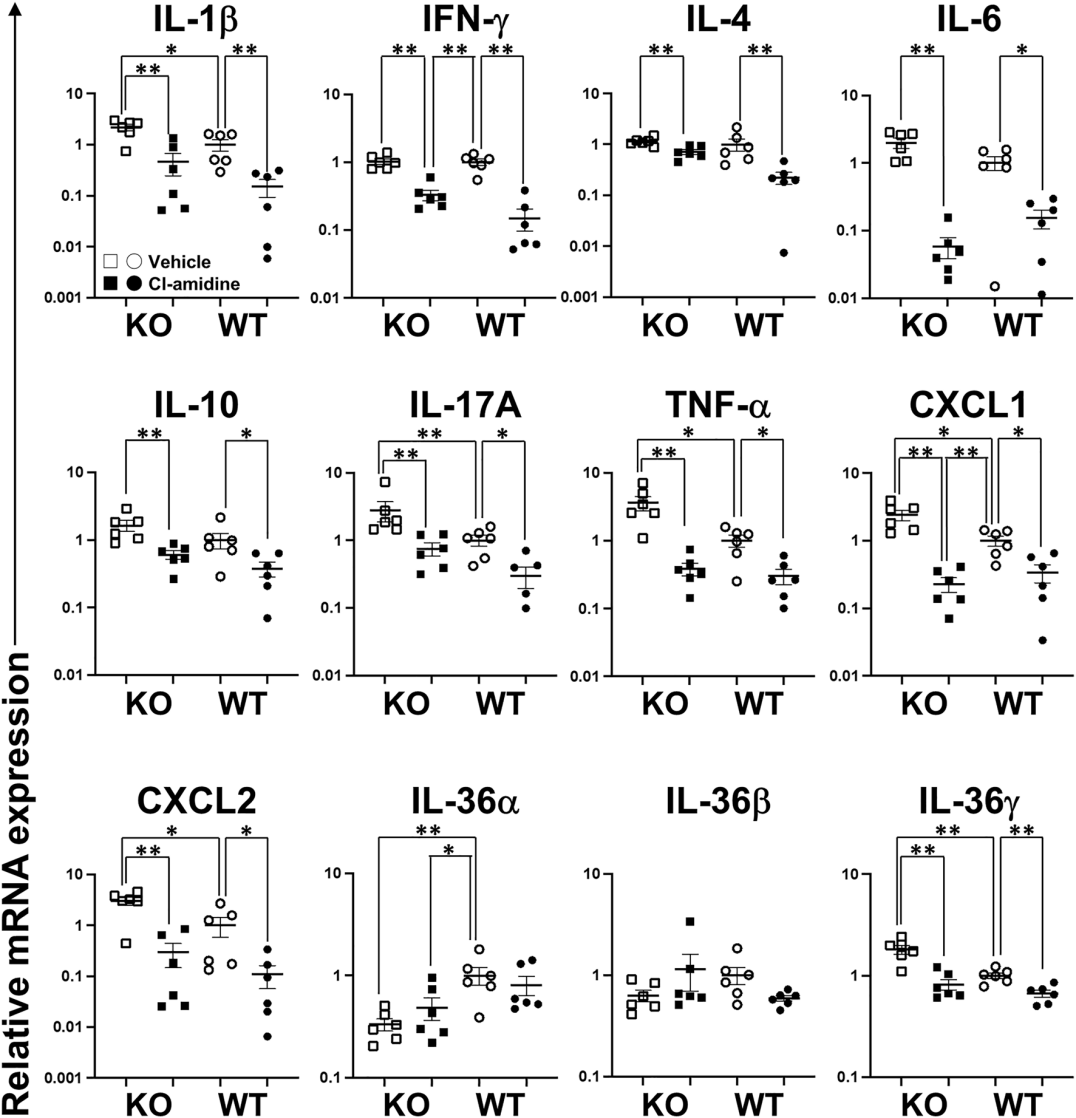
Effect of Cl-amidine on the mRNA expression levels in the skin during the contact hypersensitivity response in wild-type mice (WT) and *Il36rn*^*−/−*^ mice (KO). Relative mRNA expression of interleukin (IL)-1β, IFN-γ, IL-4, IL-6, IL-10, IL-17A, tumor necrosis factor alpha (TNF-α), C-X-C motif chemokine ligand (CXCL) 1, CXCL2, IL-36α, IL-36β, and IL-36γ was quantified using real-time reverse transcription polymerase chain reaction and normalized to endogenous glyceraldehyde-3-phosphate dehydrogenase mRNA expression levels. All values represent the mean ± SEM obtained from six mice per group (*p < 0.05, **p < 0.01).

Lastly, we examined the expression levels of several cytokines, chemokines, and growth factors during the CHS response using reverse transcription polymerase chain reaction (RT-PCR) to assess how Cl-amidine treatment inhibited the CHS response and inflammation. In agreement with findings from previous reports^14^, at 48 h after a DNFB challenge, the mRNA expression levels of IL-1β, IL-17A, tumor necrosis factor alpha (TNF-α), C-X-C motif chemokine ligand (CXCL) 1, CXCL2, and IL-36γ in the ear tissue samples of *Il36rn*^*−/−*^ mice were significantly higher than those in the ear tissue samples of wild-type mice (Fig. 4). In contrast, the mRNA level of IL-36α decreased in *Il36rn*^*−/−*^ mice in comparison with that in wild-type mice, and the loss of IL-36Ra expression did not affect the mRNA expression of interferon gamma (IFN-γ), IL-4, IL-6, IL-10, or IL-36β compared to that in wild-type mice, as previously reported^14^. In addition, in *Il36rn*^*−/−*^ mice, the mRNA expression levels of IL-1β, IFN-γ, IL-4, IL-6, IL-10, IL-17A, TNF-α, CXCL1, CXCL2, and IL-36γ decreased significantly in response to Cl-amidine treatment (Fig. 4). No significant differences were observed between the Cl-amidine treatment and control groups regarding the expression levels of IL-36α and IL-36β (Fig. 4). In wild-type mice, the mRNA expression levels of IL-1β, IFN-γ, IL-4, IL-6, IL-10, IL-17A, TNF-α, CXCL1, CXCL2, and IL-36γ reduced significantly in response to Cl-amidine treatment (Fig. 4). No significant differences in the IL-36α and IL-36β mRNA expression levels were observed between the Cl-amidine-treated and untreated groups (Fig. 4). Thus, IL-36Ra deficiency modified the mRNA expression levels of cytokines, chemokines, and growth factors at 48 h after the challenge, and Cl-amidine treatment suppressed the increase in mRNA expression levels.

## Discussion

The findings in this study illustrated that the CHS response is enhanced in *Il36rn*^*−/−*^ mice (Fig. 1). Histopathological examination revealed that inflammatory cell infiltration and the area of NETs increased significantly in *Il36rn*^*−/−*^ mice in comparison with those in wild-type mice (Figs. 2 and 3). Moreover, we observed a significant increase in the expression of various inflammatory cytokines and chemokines in *Il36rn*^*−/−*^ mice in comparison with that in wild-type mice (Fig. 4). Administration of Cl-amidine, a PAD inhibitor, attenuated the CHS response in both *Il36rn*^*−/−*^ and wild-type mice (Fig. 1). Furthermore, the number of infiltrating inflammatory cells, area of NETs, and cytokine and chemokine expression levels in CHS lesions decreased in both *Il36rn*^*−/−*^ and wild-type mice compared to those in the vehicle control (Figs. 2, 3, 4). Collectively, we found that under the CHS response, NET production was enhanced in *Il36rn*^*−/−*^ mice compared to that in wild-type mice, and the inhibition of NET formation suppressed the exacerbation of CHS. Our study is the first to suggest the involvement of NETs in the immune responses in CHS.

**Figure 5:**
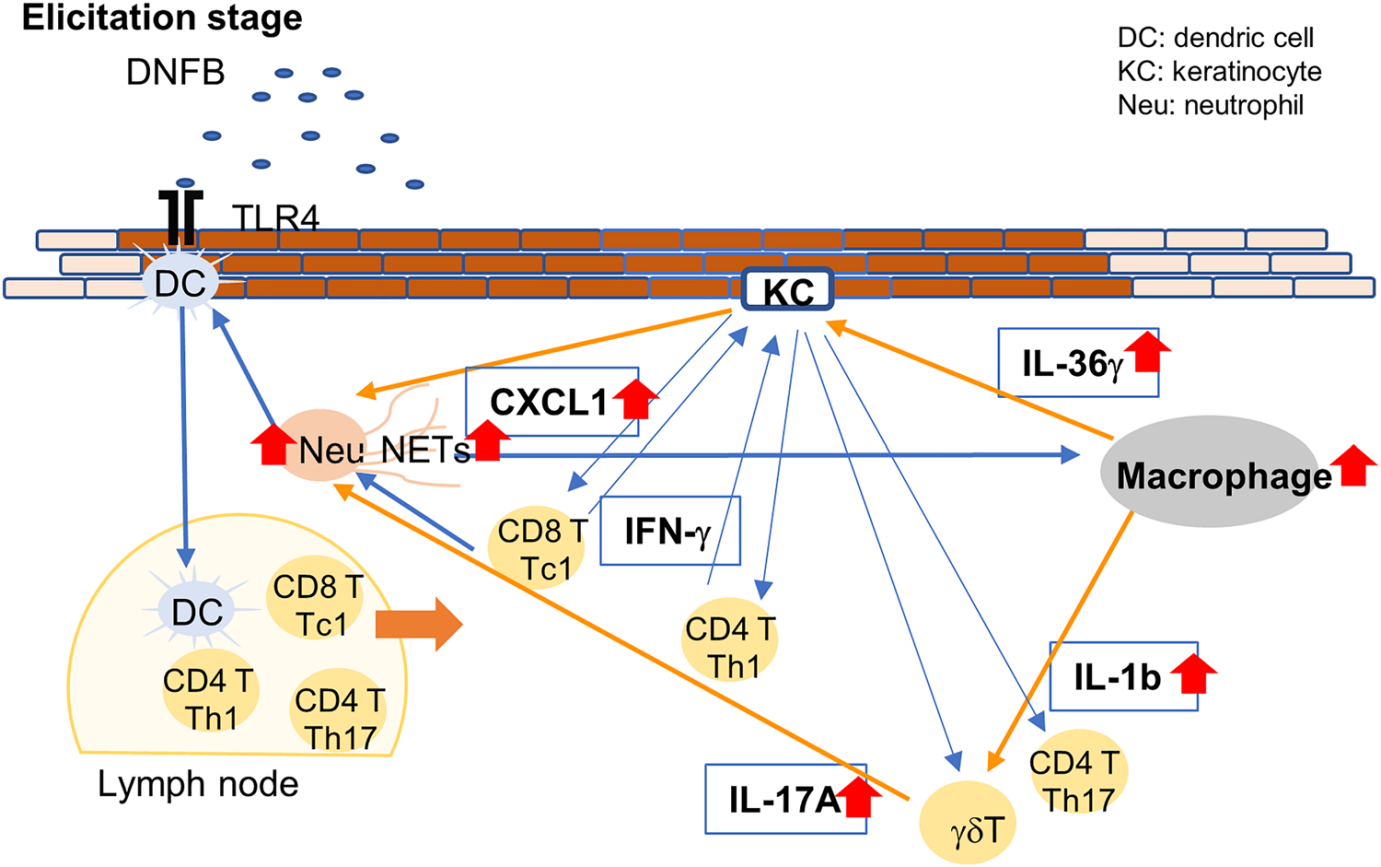
Scheme illustrating the pathology of contact hypersensitivity lesions in the elicitation phase. In *Il36rn*^−/−^ mice, the enhancement of NET formation promoted the production of cytokines, such as interleukin (IL)-36γ and IL-1β, by macrophages. IL-1β may induce IL-17A production from γδ T cells and contribute to neutrophil generation. In addition, IL-36γ stimulation may induce keratinocyte activation, and C-X-C motif chemokine ligand (CXCL) 1 produced by keratinocytes may induce the migration of neutrophils, CD4^+^ T cells, CD8^+^ T cells, and γδ T cells, further promoting neutrophil generation. KC, keratinocyte; DC, dendritic cell; NETs, neutrophil extracellular traps; Neu, neutrophils. γδ T: γδ T cells.

The number of macrophages, CD4^+^ T cells, and CD8^+^ T cells was significantly higher in *Il36rn*^*−/−*^ mice than in wild-type mice. Consistent with the pathological findings, *Il36rn*^*−/−*^ mice showed significantly high levels of inflammatory cytokines (including IL-1β, CXCL1, and CXCL2), Th17/Tc17 cytokines (including IL-17A and TNF-α), and IL-36γ (Figs. 1, 2, 3). Based on these results and findings from previous reports, we propose a novel model of the role of NETs in CHS elicitation (Fig. 5). It is known that in contact dermatitis, neutrophils activate T cells by inducing DC maturation during antigen presentation in the sensitization and elicitation phases^17^. IL-17 is produced by γδ T cells in the skin during the process^18^. During elicitation, CD8^+^ tissue-resident memory T cells trigger a massive neutrophil infiltration in the epidermis within 12 h of re-exposure to contact allergens^19^. In addition, activated Th1 and CD8^+^ T cells (Tc1) produce IFN-γ^17^, which acts on keratinocytes to generate Th1 cytokines and lead to the infiltration of inflammatory cells into the skin during the effector phase^17^. As NET formation was confirmed in this study, NETs may contribute to the CHS response. NETs have been reported to promote macrophage pyroptosis and macrophage-derived IL-1β production^20^. In addition, reportedly, macrophages secrete IL-36^21^. Furthermore, in an in vitro experiment, IL-36γ induced CXCL1 production via the activation of keratinocytes and promoted the migration of neutrophils, αβT cells, and γδT cells^22^. Therefore, enhanced NET formation in *Il36rn*^*−/−*^ mice may promote macrophage recruitment and increase IL-1β and IL-36γ production. IL-1β may induce the production of IL-17A in γδ T cells and contribute to the production of neutrophils. In addition, IL-36γ stimulation may induce keratinocyte proliferation, and CXCL1 generated by keratinocytes may induce the migration of neutrophils, CD4^+^ T cells, CD8^+^ T cells, and γδ T cells, leading to a subsequent increase in neutrophil and NET production.

Treatment with Cl-amidine, an inhibitor of NET formation, suppressed the CHS response in both *Il36rn*^*−/−*^ and wild-type mice (Fig. 1), suggesting that NETs could be a potential therapeutic target for CHS. Currently, steroids are the primary treatment agent used in allergic contact dermatitis^23^. However, steroids can increase susceptibility to infections, such as scabies incognito/crusted scabies, tinea incognito, impetigo incognito, candidiasis, herpes incognito, and demodicidiosis^24^. Steroids target NF-κB^25–27^; this could be a possible reason why steroids block a wide range of immune defenses. Therefore, the use of Cl-amidine may possibly lead to a favorable side effect profile, such as a narrower range of treatment targets and a lower effect on the immune response. Regarding pathological findings, both *Il36rn*^*−/−*^ and wild-type mice showed the inhibition of not only the increase in NET area, but also the increase in neutrophil, macrophage, CD4^+^ T cell, and CD8^+^ T cell production (Figs. 2 and 3). Consistent with the pathological findings, the increase in the levels of inflammatory cytokines (such as IL-1β, CXCL1, and CXCL2), Th1/Tc1 cytokines (such as IFN-γ), Th2/Tc2 cytokines (such as IL-4 and IL-10), Th17/Tc17 cytokines (such as IL-6, IL-17A, and TNF-α), and IL-36γ (Fig. 4) was inhibited in both *Il36rn*^*−/−*^ and wild-type mice, as evaluated using RT-PCR. Conversely, when we compared the levels of cytokines that were inhibited by Cl-amidine between Cl-amidine-treated *Il36rn*^*−/−*^ mice and untreated wild-type mice, differences were observed in the levels of IFN-γ and CXCL1, but not in those of inflammatory cytokines, such as IL-1β and CXCL2; Th2/Tc2 cytokines, such as IL-4 and IL-10; Th17/Tc17 cytokines, such as IL-6, IL-17A, and TNF-α; and IL-36γ cytokines (Fig. 4). Collectively, the blockade of NET formation by Cl-amidine may serve as a novel treatment option for allergic contact dermatitis.

Conversely, the comparison of cytokine expression in *Il36rn*^*−/−*^ mice treated with Cl-amidine and untreated wild-type mice revealed differences in the IFN-γ and CXCL1 levels, and a completely similar recovery was not observed in the two groups, suggesting that the pathophysiology of contact dermatitis exacerbation in IL-36Ra knockout mice is not related solely to NETs.

This study has a limitation that could be addressed in future studies. Since all samples in this experiment were obtained after elicitation, the pathophysiology was observed only during the elicitation phase. However, the reaction process of contact dermatitis can be roughly divided into the sensitization and elicitation phases. Therefore, to determine the effect of NETs on the pathophysiology of contact dermatitis during sensitization, it is necessary to examine the percentage of naïve, activated, and memory T cells in regional lymph nodes and tissues after sensitization. Additional experiments using flow cytometry should be conducted in the future. In a sepsis model, mice treated with Cl-amidine were reported to have higher IL-10 levels in the plasma than in mice not treated with Cl-amidine^28^; however, in our experiments, we found no difference in IL-10 levels in the plasma (data not shown). The fact that, in our study, IL-10 levels differed in the ear tissue but not in the plasma was presumably because CHS is a localized inflammation and the inflammation was not high enough to affect plasma IL-10 levels.

In conclusion, the results of our study suggest that although CD4^+^ T cells, CD8^+^ T cells^29^, and neutrophils^14,18,30,31^ have been implicated in the immune reactions in contact dermatitis, NETs may also exacerbate immune reactions in CHS. Therefore, the inhibition of NET formation may be a new therapeutic strategy in contact dermatitis.

## Methods

### Mice

The study was approved by the Regulations for the Management of Laboratory Animals at Fujita Health University, and all procedures were conducted in accordance with the guidelines of the Animal Care and Use Committee at Fujita Health University (APU19017). Our study with observational experimental design was carried out in compliance with the ARRIVE guidelines. We bred and housed the mice in a specific pathogen-free barrier facility. *Il36rn*^*−/−*^ mice were generated on a C57BL/6NCr1 background, as previously reported^32^, and the genotype was confirmed by allele-specific PCR. Wild-type mice (C57BL/6NCr1) were obtained from Charles River Laboratories (Inc., Wilmington, MA, USA). Gender-matched female mice, aged 8 to 12 weeks, were used in these experiments.

### CHS and Cl-amidine treatment

We created the CHS induction mouse model in *Il36rn*^*−/−*^ and wild-type mice and assigned them to Cl-amidine treatment and no treatment groups, respectively. Six mice were assigned to each group. We generated the CHS induction mouse model using DNFB (Wako Pure Chemicals, Tokyo, Japan), as previously reported^33^. On day 0, the mice were sensitized by applying 25 µL of 0.5% DNFB in acetone/olive oil (4:1) on the shaved dorsal surface and were then topically challenged with 15 µL of 0.2% DNFB in acetone/olive oil (4:1) on each side of both ears on day 5. To investigate the effect of Cl-amidine during the CHS response, *Il36rn*^*−/−*^ and wild-type mice were intraperitoneally injected with 10 mg/kg Cl-amidine (Cayman Chemical, MI, USA) or an equal volume of vehicle (dimethyl sulfoxide solution) on days 0–5. We measured the ear swelling using dial thickness gauges (Peacock, Ozaki MFG. Co., Ltd, Chiba, Japan) before the challenge and after 24 and 48 h. Measurements for each ear were performed three times, and the average value was used for analysis. We collected ear tissue samples on day 7 for RT-PCR and histopathological analysis.

### Histological staining

We excised the ears of mice in each group 48 h after the DNFB challenge. The ear tissues were fixed in 4% paraformaldehyde and embedded in paraffin, and 6 µm sections were prepared. We stained the preparations with hematoxylin and eosin (H&E) and counted the number of MPO-positive neutrophils, F4/80-positive macrophages, CD4-positive T cells, and CD8-positive T cells in 10 high-power fields (0.16 mm^2^; magnification,  400×). Two investigators, who were blinded to the study, independently examined each section. The mean values of these measurements were utilized for analysis.

### Immunohistochemical staining

Paraffin-embedded tissues were cut into 4 μm sections, deparaffinized using xylene, permeabilized with ethanol, and rehydrated in phosphate-buffered saline (PBS). The deparaffinized sections were treated with an endogenous peroxidase blocking solution (horse serum diluted at a 1:1 ratio in PBS + 1% bovine serum albumin) for 20 min at 27 °C. The sections were treated overnight with specific primary antibodies against F4/80 (1:800, Cell Signaling Technology, Inc., Tokyo, Japan), CD3 (1:500, Cell Signaling Technology, Inc.), CD4 (1:200, Cell Signaling Technology, Inc.), and CD8 (1:800, Cell Signaling Technology, Inc.) at 4 °C, followed by three washes with PBS. Secondary antibodies conjugated to biotin were then applied and incubated with an avidin–biotin complex (Vector Laboratories: VECTASTAIN Elite ABC Kit #PK-6101) for 30 min at 27 °C, followed by washing three times with PBS. Peroxidase activity was observed with the use of an ImmPACT DAB substrate kit (Vector Laboratories: #SK-4105). The slides were counterstained with hematoxylin. For the negative control, the sections were not treated with primary antibodies.

### Immunofluorescence staining

The skin of the ear samples was cut into 4 μm-thick sections, and immunofluorescence staining was performed to determine NET release utilizing antibodies against citrullinated-histone H3 and MPO, as published previously^15^. The ear sections were deparaffinized in xylene, permeabilized with ethanol, rehydrated in PBS, and blocked with a blocking solution (10% donkey serum and 1% bovine serum albumin) for 30 min at 27 °C. Next, the sections were treated with specific primary antibodies against citrullinated histone H3 (1:250, Abcam, Cambridge, UK) and MPO (1:100, Research and Diagnostic Systems, Inc., Minneapolis, MN, USA) for 2 h at 27 °C. After washing three times with PBS, the slides were treated with secondary antibodies (donkey anti-goat immunoglobulin G (IgG), Novus Biologicals, Littleton, CO, USA; Alexa Fluor 488, Thermo Fisher Scientific, Waltham, MA, USA; donkey anti-rabbit IgG, Novus Biologicals; and Alexa Fluor 647, Thermo Fisher Scientific) for 30 min at 27 °C. The sections were washed three times with PBS and then enclosed in a medium containing 4′,6-diamidino-2-phenylindole (Abcam). Confocal imaging was performed with the use of an Olympus Fluoview 1000 microscope (Olympus Life Sciences, Tokyo, Japan). The NETs showed positive staining for both MPO and citrullinated histone H3. Relative NETs area was calculated as previously reported^34,35^. Briefly, we randomly selected five sections per mouse and then used the Image J software to add the pixels in the area of NETs in each section. Thereafter, we averaged the number of pixels per section per mouse. The average NETs area for the six mice in the no-treatment group of wild type mice was then taken as 1, and for the other groups, they were calculated as ratios. Two investigators independently examined each section in a blinded manner, and the mean values of the measurements were utilized for analysis.

### RNA isolation and RT-PCR

Total RNA was extracted from the ear tissue samples with Qiagen RNeasy spin columns (Qiagen, Crawley, UK). Total RNA was reverse-transcribed into cDNA with the use of the Prime Script RT Reagent Kit (Takara Bio Inc., Shiga, Japan) in accordance with the manufacturer’s instructions. The expression levels of IL-1β (Mm.PT.58.41616450), IFN-γ (Mm.PT.58.41769240), IL-4 (Mm.PT.58.32703659), IL-6 (Mm.PT.58.10005566), IL-10 (Mm.PT.58.13531087), IL-17A (Mm.PT.58.6531092), TNF-α (Mm.PT.58.12575861), CXCL1 (Mm.PT.58.42076891), CXCL2 (Mm.PT.58.1045839), IL-36α (Mm.PT.58.12651602), IL-36β (Mm.PT.58.11528127), and IL-36γ (Mm.PT.58.30810984) were quantified by RT-PCR on a Light Cycler System (F. Hoffmann-La Roche, Ltd., Basel, Switzerland). The PCR samples were dispensed in microcapillary tubes, with a reaction volume of 20 µL, containing 2.0 µL of the diluted cDNA solution, and the PCR program was conducted in accordance with the manufacturer’s instructions. Glyceraldehyde-3-phosphate dehydrogenase (*GAPDH*; Mm.PT.39a.1) was utilized as an internal control. The relative mRNA expression levels of various target genes normalized to the expression level of *GAPDH* were calculated using the 2^−∆∆Ct^ method. The primer sequences utilized for each gene were chosen by pre-validated PrimeTime qPCR assays (Integrated DNA Technologies, Coralville, IA, USA).

### Statistical analysis

Data were analyzed using GraphPad Prism software version 7 (GraphPad Software, La Jolla, CA, USA) and expressed in terms of mean ± SEM. The Mann–Whitney *U* test was used for determining the level of significance of differences between samples, and the Bonferroni’s test was used for multiple comparisons. Statistical significance was set at *p* < 0.05.

## Supplementary Information

Supplementary Information.

Supplementary Figure 1.

## Data Availability

The datasets generated and/or analyzed during the current study are available from the corresponding author upon reasonable request.
