# Supplementary material for: Neutrophil extracellular traps are involved in enhanced contact hypersensitivity response in IL-36 receptor antagonist-deficient mice
Source: Sci Rep. 2022 Aug 4;12:13384. doi: 10.1038/s41598-022-16449-z (PMC9352770; doi:10.1038/s41598-022-16449-z)
Supplement: Supplementary file 1 — Supplementary Information. [file 41598_2022_16449_MOESM1_ESM.docx]

**SUPPLEMENTAL MATERIAL**

**Neutrophil extracellular traps are involved in enhanced contact hypersensitivity response in IL-36 receptor antagonist-deficient mice**

Yurie Hasegawa^1^, Yohei Iwata^1^, Hidehiko Fukushima^1^, Yoshihito Tanaka^1^, Soichiro Watanabe^1^, Kenta Saito^1^, Hiroyuki Ito^1^, Mizuki Sugiura^1^, Masashi Akiyama^2^ & Kazumitsu Sugiura^1*^

Affiliations :

^1^ Department of Dermatology, Fujita Health University School of Medicine, 1-98 Dengakugakubo, Kutsukake-cho, Toyoake, Aichi 470-1192, Japan

^2^ Department of Dermatology, Nagoya University Graduate School of Medicine, 65 Tsurumai-cho, Showa-ku, Nagoya, Aichi 466-8550, Japan

Corresponding author:

Dr. Kazumitsu Sugiura

E-mail: ksugiura@fujita-hu.ac.jp

**Supplementary Methods**

***In vitro* experiments**

*In vitro* experiments and immunocytochemical staining were performed to evaluate the effects of Cl-amidine on neutrophils and NETs using antibodies against citrullinated histone H3 and MPO as previously reported [1].

Neutrophils (1×10^6^) were placed on sterile round glass coverslips in 6-well cell culture plates and incubated for 30 min at 37 °C in a 5% CO2 incubator. For 30 min, 200 M Cl-amidine or PBS were prepped in a 5% CO_2_ incubator at 37 °C. The cells were then treated with 100 M PMA or PBS and incubated at 37 °C for 4 h.

**Immunocytochemistry staining**

After carefully removing glass coverslips with adherent cells from the 6-well culture plates, the cells were fixed in 10% formalin and blocked with a blocking solution (10% donkey serum and 1% bovine serum albumin). Thereafter, the adherent cells were treated with specific primary antibodies against citrullinated histone H3 (1:300, Abcam, Cambridge, UK) and MPO (1:300, Research and Diagnostic Systems, Inc., Minneapolis, MN, USA) for overnight at 4 °C and subsequently with secondary antibodies (1:500, donkey anti-goat immunoglobulin G (IgG), Novus Biologicals, Littleton, CO, USA; Alexa Fluor 488, Thermo Fisher Scientific, Waltham, MA, USA; 1:500, donkey anti-rabbit IgG, Novus Biologicals; and Alexa Fluor 647, Thermo Fisher Scientific) for 60 min at 27 °C. The glass coverslips were then enclosed in a medium containing 4′,6-diamidino-2-phenylindole (Abcam). Confocal imaging was performed with the use of an Olympus Fluoview 1000 microscope (Olympus Life Sciences, Tokyo, Japan). For quantification of neutrophils (MPO-stained), image data were imported into the ImageJ software. Ten pictures were randomly collected from different positions on the glass coverslips and the mean value per picture was determined.

For NET measurement, ten pictures were randomly obtained from different positions of the glass coverslips. To count the number of cells, the image files were analyzed using the ImageJ software. NETs were positive for both MPO and citrullinated histone H3 staining. The percentage of NETs formed was calculated as 100 × (number of NET/total number of cells).

**References**

1 Wang, N. *et al*. Neutrophil extracellular traps induced by VP1 contribute to pulmonary edema during EV71 infection. *Cell Death Discov.* **5,** 111; 10.1038/s41420-019-0193-3 (2019).

**Supplementary Figure Legends**

**Supplementary figure S1.** **Effect of Cl-amidine on neutrophils and neutrophil extracellular trap (NET) formation *in vitro***

We investigated whether Cl-amidine is directly involved in the reduction of neutrophils. Neutrophils isolated from the abdominal cavity of mice were treated with Cl-amidine or PBS. Myeloperoxidase (MPO)-positive neutrophils when treated with Cl-amidine showed no difference (*p* < 0.005) from MPO-positive neutrophils not treated with Cl-amidine. This result indicates that Cl-amidine does not directly reduce the number of neutrophils. (a) Representative immunocytochemistry image of MPO^+^ neutrophils (red). Scale bar = 50 μm. (b) Quantification of MPO^+^ neutrophils with and without Cl-amidine therapy. With a *t*-test *p* > 0.05, the average of the number of MPO^+^ neutrophils in 10 sections is depicted in the histograms.

We further evaluated the effect of Cl-amidine on NET depletion *in vitro*, as well. Neutrophils isolated from the abdominal cavity of mice were pretreated with Cl-amidine or PBS and then NETs induced with phorbol myristate acetate (PMA) were observed. Consistent with *in vivo* results, Cl-amidine inhibited NETs release after 4 h of culture compared with that in untreated neutrophils (48.07 ± 1.093 vs. 6.829 ± 1.243, ***p* < 0.01). This result indicates that Cl-amidine suppresses NETs release *in vitro*, as well. (c) Representative immunocytochemistry image of NET structures. Arrow heads show the formation of NETs that were MPO-positive (red) and citrullinated histone-positive (green). The nuclei were counterstained with 4′,6-diamidino-2-phenylindole (blue). Scale bar = 50 μm. (d) Quantification of NETs in neutrophils induced with PMA under Cl-amidine and no-treatment conditions. The histograms show the mean % SEM of NETs producing neutrophils, with *t*-test ***p* < 0.01.
