# Supplementary figures and images for: Neutrophil extracellular traps are involved in enhanced contact hypersensitivity response in IL-36 receptor antagonist-deficient mice
Source: Sci Rep. 2022 Aug 4;12:13384. doi: 10.1038/s41598-022-16449-z (PMC9352770; doi:10.1038/s41598-022-16449-z)

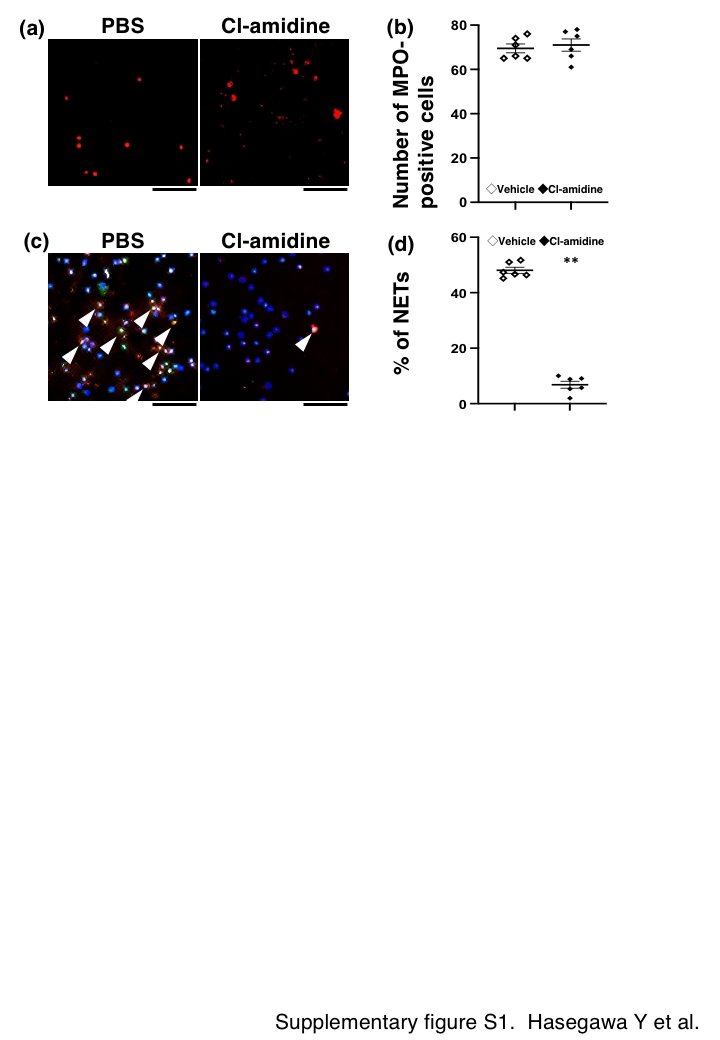

Supplement: Supplementary file 2 — Supplementary Figure 1. [file 41598_2022_16449_MOESM2_ESM.tiff]
